# Supplementary material for: A Critical View of the Application of the APEX Software (Aqueous Photochemistry of Environmentally-Occurring Xenobiotics) to Predict Photoreaction Kinetics in Surface Freshwaters
Source: Molecules. 2019 Dec 18;25(1):9. doi: 10.3390/molecules25010009 (PMC7017383; doi:10.3390/molecules25010009)
Supplement: Supplementary file 1 [file molecules-25-00009-s001.zip › 3.2.4_gcc-4.4.0.zip!/3.2.4_gcc-4.4.0/3.2.4_gcc-4.4.0/doc/octave/html/interpreter/Nonlinear-Programming.html]

Nonlinear Programming - Untitled


Next: Linear Least Squares,
Previous: Quadratic Programming,
Up: Optimization


---

### 24.3 Nonlinear Programming

Octave can also perform general nonlinear minimization using a
successive quadratic programming solver.

— Function File: [x, obj, info, iter, nf, lambda] = **sqp** (x, phi, g, h, lb, ub, maxiter, tolerance)  
> Solve the nonlinear program
>
> ```
>                min phi (x)
>                 x
> ```
>
> subject to
>
> ```
>                g(x)  = 0
>                h(x) >= 0
>                lb <= x <= ub
> ```
>
> using a successive quadratic programming method.
>
> The first argument is the initial guess for the vector x.
>
> The second argument is a function handle pointing to the objective
> function. The objective function must be of the form
>
> ```
>                y = phi (x)
> ```
>
> in which x is a vector and y is a scalar.
>
> The second argument may also be a 2- or 3-element cell array of
> function handles. The first element should point to the objective
> function, the second should point to a function that computes the
> gradient of the objective function, and the third should point to a
> function to compute the hessian of the objective function. If the
> gradient function is not supplied, the gradient is computed by finite
> differences. If the hessian function is not supplied, a BFGS update
> formula is used to approximate the hessian.
>
> If supplied, the gradient function must be of the form
>
> ```
>           g = gradient (x)
> ```
>
> in which x is a vector and g is a vector.
>
> If supplied, the hessian function must be of the form
>
> ```
>           h = hessian (x)
> ```
>
> in which x is a vector and h is a matrix.
>
> The third and fourth arguments are function handles pointing to
> functions that compute the equality constraints and the inequality
> constraints, respectively.
>
> If your problem does not have equality (or inequality) constraints,
> you may pass an empty matrix for cef (or cif).
>
> If supplied, the equality and inequality constraint functions must be
> of the form
>
> ```
>           r = f (x)
> ```
>
> in which x is a vector and r is a vector.
>
> The third and fourth arguments may also be 2-element cell arrays of
> function handles. The first element should point to the constraint
> function and the second should point to a function that computes the
> gradient of the constraint function:
>
> ```
>                           [ d f(x)   d f(x)        d f(x) ]
>               transpose ( [ ------   -----   ...   ------ ] )
>                           [  dx_1     dx_2          dx_N  ]
> ```
>
> The fifth and sixth arguments are vectors containing lower and upper bounds
> on x. These must be consistent with equality and inequality
> constraints g and h. If the bounds are not specified, or are
> empty, they are set to -realmax and realmax by default.
>
> The seventh argument is max. number of iterations. If not specified,
> the default value is 100.
>
> The eighth argument is tolerance for stopping criteria. If not specified,
> the default value is eps.
>
> Here is an example of calling `sqp`:
>
> ```
>           function r = g (x)
>             r = [ sumsq(x)-10;
>                   x(2)*x(3)-5*x(4)*x(5);
>                   x(1)^3+x(2)^3+1 ];
>           endfunction
>           
>           function obj = phi (x)
>             obj = exp(prod(x)) - 0.5*(x(1)^3+x(2)^3+1)^2;
>           endfunction
>           
>           x0 = [-1.8; 1.7; 1.9; -0.8; -0.8];
>           
>           [x, obj, info, iter, nf, lambda] = sqp (x0, @phi, @g, [])
>           
>           x =
>           
>             -1.71714
>              1.59571
>              1.82725
>             -0.76364
>             -0.76364
>           
>           obj = 0.053950
>           info = 101
>           iter = 8
>           nf = 10
>           lambda =
>           
>             -0.0401627
>              0.0379578
>             -0.0052227
> ```
>
> The value returned in info may be one of the following:
>
> 101: The algorithm terminated because the norm of the last step was less than `tol * norm (x))` (the value of tol is currently fixed at `sqrt (eps)`—edit sqp.m to modify this value. 102: The BFGS update failed. 103: The maximum number of iterations was reached (the maximum number of allowed iterations is currently fixed at 100—edit sqp.m to increase this value).
>
>
>
>
> **See also:** qp.
